# Comparing the accuracy of PCR-capillary electrophoresis and cuticle microhistological analysis for assessing diet composition in ungulates: A case study with Pyrenean chamois

**DOI:** 10.1371/journal.pone.0216345

**Published:** 2019-05-22

**Authors:** Johan Espunyes, Carme Espunya, Sara Chaves, Juan Antonio Calleja, Jordi Bartolomé, Emmanuel Serrano

**Affiliations:** 1 Wildlife Ecology & Health Group (WE&H) and Servei d’Ecopatologia de Fauna Salvatge (SEFaS), Departament de Medicina i de Cirurgia Animals, Universitat Autònoma de Barcelona UAB, Bellaterra, Spain; 2 Departament de Bioquímica i de Biologia Molecular, UAB, Bellaterra, Spain; 3 Centre de Recerca Ecològica i Aplicacions Forestals (CREAF), Cerdanyola del Vallès, Spain; 4 Grup de Recerca en Remugants, Departament de Ciència Animal i dels Aliments, UAB, Bellaterra, Spain; 5 Dipartimento di Scienze Veterinarie, Universitá di Torino, Grugliasco, Torino, Italy; Austrian Federal Research Centre for Forests BFW, AUSTRIA

## Abstract

The study of diet composition is required to understand the interactions between animal and plant ecosystems. Different non-invasive techniques applied on faecal samples have commonly been used for such purposes, with cuticle microhistological analysis (CMA) and emerging DNA-based methods being the most relevant. In this work, we refined and optimized a qualitative DNA-based approach combining PCR amplification of long *trnL(UAA) and ITS2* fragments and capillary electrophoresis (PCR-CE), instead of short *trnL(UAA)* fragments and massive sequencing technologies commonly reported. To do so, we developed a controlled diet assay using a stabled Pyrenean chamois specimen (*Rupicapra pyrenaica pyrenaica*), which included representative herbaceous and shrubby plant species. We also assessed the impact of sample freshness on the diet determination of this mountain caprinae by exposing faecal samples to the outdoor environment for three weeks. Faecal samples from both experiments were analysed by qualitative PCR-CE and semi-quantitative CMA in order to compare the pros and cons of both approaches. Our results show that all of the offered plant species were detected by both methodologies although CMA over-detected shrubs compared to herbaceous species. At the same time, sample degradation due to sustained climate exposure is a limiting factor for molecular analysis, but not for CMA. Taken all together, our results suggest that the qualitative information obtained by CMA and PCR-CE can be interchangeable when faecal samples are fresh (less than one week after deposition) but, afterwards, molecular analysis underestimates diet composition probably due to DNA degradation. CMA, however, can accurately be used at least three weeks after defecation. Moreover, by combining the results of simultaneous PCR amplification of two complementary genes, this optimized PCR-CE methodology provides a reliable, feasible and more affordable alternative for multiple and routine analyses of complex samples. Neither CMA nor PCR-CE seems to solve comprehensively the quatification of herbivore diets and thus further research needs to be done.

## Introduction

Food selection is a central concept in wildlife nutrition studies [1]. Although food intake evaluation is the starting point for any study of animal nutrition, field ecologists struggle with the challenging task of assessing diet composition. Feeding strategies of free-ranging species are not only influenced by foraging behaviour, food quality and availability but also by the individuals’ requirements for reproduction and maintenance [2]. Large herbivores play a major role in forming the shapes and functions of terrestrial ecosystems as they exert an impact on landscape dynamics and biodiversity [3]. Comprehensive data on their feeding habits are crucial to understand their influence on the ecology of other organisms as they drive the viability of plant communities [4], modify nutrient cycles [5] and regulate carnivore populations [6]. On the other hand, this information is necessary to evaluate their impact on threatened flora [7] and hence to elaborate plans for plant protection [8] or population control [9]. Therefore, knowing the diet composition of herbivores is an essential step towards building an ecological model of plant-herbivore interactions and is basic to many aspects of wildlife and plant research and conservation [10,11]. In fact, modern diet analysis has improved the understanding of dietary niche partitioning and the coexistence of similar herbivores [12,13] as well as enhanced conservation strategies, for example, for endangered woodland caribous [14].

Along these lines, non-invasive techniques for the analysis of herbivore diets (e.g. faeces collection or animal observation) are broadly established since they do not imply animal culling. The simplest procedures are the *in situ* observation of herbivore ingests of available vegetation, and the time-lapse visualization of chewed plants [15]. However, these approaches are both highly time-consuming because extensive and representative geographical areas need to be monitored in order to obtain conclusive information. Alternatively, information on diet composition can be inferred from the corresponding herbivore faeces through the analysis of plant material remaining after digestion. Two effective faeces-related methodologies should be noted. The first, cuticle microhistological analysis (CMA), is a traditional and well-established method that can provide reliable semi-quantitative data through the identification of plant cell structures, mainly the epidermis and trichomes, visualised under an optical microscope [16–18]. CMA has been extensively applied to different types of complex samples, including faeces, rumen contents and even coprolites [19], due to the strong resistance of plant epidermal cuticles to climate and environmental degradation processes. However, CMA presents important drawbacks that limit its use. Significant expertise in microscopic skills is required [20]. In addition, identification of plant structures is frequently impaired by the strong similarity of anatomic structures between taxonomically related genera to the extent that in some cases only the family level can be determined [18].

The second category of faeces-related methodologies involves the analysis of remaining plant genomic DNA isolated from faeces, also known as DNA barcoding methods [21–23]. DNA barcoding is considered a powerful and accurate alternative to morphological methods and has been applied in many biological studies including the authentication of herbal medicinal products [24,25], ancient permafrost sediment analysis [26–28], and the assessment of herbivore diet composition [29–34]. Next-generation sequencing (NGS) technology, is currently the most used DNA barcoding method and consists of the identification of different taxa using marker gene sequences, also called barcodes, through amplification by PCR and subsequent sequencing by massive NGS [22,35–38]. In plants, different chloroplast genes have been extensively used—e.g. *matK*, *trnL(UAA)*, *rbcL* and *trnH-psbA*—either alone or combined with nuclear genes, such as the Internal Transcribed Spacer regions of ribosomal DNA, *ITS1* and *ITS2* [36,39–41]. However, NGS-DNA barcoding implementation has been hampered by the economic cost of sequencing technologies [35,38], whereas an alternative method involving capillary electrophoresis has emerged as a more affordable and suitable option for multiple and routine analysis of complex samples [32,42]. This alternative technique, hereafter called PCR-capillary electrophoresis (PCR-CE), applies capillary electrophoresis to determine the amplicons lengths, instead of DNA-sequencing as in NGS-DNA barcoding. We obtain a migration pattern that allows the detection of several peaks characteristic of multi-species samples. The subsequent botanical adscription of each peak is realized through matching experimental data with those available in the GenBank database [43]. PCR-CE is not commonly used in studies of diet composition despite being fast, inexpensive and frequently applied in the food industry ([44,45], but see [32]). Usually, the resolution of this method is considered low for complex samples because plant species with similar fragment sizes can account for the same peak due to a visual overlap [32]. However, this drawback can be overcome by combining simultaneous PCR amplification of two complementary genes [42].

Here, we wanted to refine and optimize a PCR-CE analysis for a higher resolution in the determination of diet composition. For this purpose, we subjected a stabled chamois specimen to a controlled diet, which included representative herbaceous plants and woody shrubs of the eastern Pyrenees ecosystem. We developed a method combining simultaneous PCR amplification of two genes, *trnL(UAA)* and *ITS2*, and capillary electrophoresis to determine the corresponding fragments length. We expected this PCR-CE alternative method to led to a higher fragment resolution and more precise plant species identification compared to conventional amplifications methods previously described [46,47] avoiding, at the same time, the high cost and the need for specific expertise on NGS. Simultaneously, cuticle microhistological analysis was also carried on these samples in order to compare both technical approaches.

On the other hand, one of the major concerns in the determination of diet composition is knowing to what extent the freshness of faecal samples may compromise the obtaining of reliable results. In that way, we also aimed at determining if the time-lapse between animal deposition and faecal sampling impacts on CMA and PCR-CE achievements. As a pilot assessment, we exposed a pool of faeces obtained from hunter-harvested Pyrenean chamois (*Rupicapra pyrenaica pyrenaica* Bonaparte) to outdoor environmental conditions for several weeks. We then determined the number of plant species detected by both methodologies every week along the study. Being used in archeobotany [19], we expected CMA results not to be influenced by environmental exposure. On the other hand, due to the high degradability of DNA [48], we expected an effect of the time of exposure on the number of plants detected by the DNA-based method. At the same time, this assessment allowed us to test the efficiency of PCR-CE on a more species-diverse sample.

## Materials and methods

### Study area and sampling procedure

Fresh plant samples for the controlled diet tests were collected in June 2015 in the Freser-Setcases National Game Reserve (FSNGR), Eastern Pyrenees, Spain (42° 22’ N, 2° 08’ E). The FSNGR is a typical sub-humid subalpine and alpine bioclimatic region with a noticeable Mediterranean climatic influence. It covers an area of 20,200 ha, ranging from 1,800 to 2,910 m.a.s.l. and hosts a population of about 3,000 Pyrenean chamois [49]. The fresh faecal samples used for the time-course degradation study were collected from 12 hunter-harvested Pyrenean chamois during the hunting season of 2014–2015 in the same area. Faecal and plant samples were refrigerated at 4°C during transport to our facilities and stored frozen at -20°C until processing.

### Controlled diet test

A twelve-year-old female Pyrenean chamois was fed on several diets that overall included 13 vascular plant species, comprising herbaceous plants and woody shrubs representative of the subalpine and alpine grassland ecosystem of the FSNGR. Three different plant mixtures were consecutively offered and intercalated with a four-day exclusive *Medicago sativa* (L.) diet, as shown in Table 1. Fresh faeces were collected before beginning the controlled diet (day 7) and the day after the corresponding diet intake, and were immediately stored at -20°C until processing. The chamois used in this study was observed with ectopia lentis—an ocular condition—in the central Catalan Pyrenees (Pallars Sobirà; 42° 58’ N, 1° 22’ E). The animal was captured by hand by a team of rangers and sent to the wildlife facilities of the Universitat Autònoma de Barcelona for veterinary inspection. The chamois was maintained in an isolated box (6m x 3m) with natural light for six months, pending translocation. Even though the animal showed some degree of visual impairment, it was able to feed selectively on the offered diet (personal observations).

**Table 1:** Schedule of the controlled diet offered to a stabled specimen of Pyrenean chamois to compare the effectiveness of cuticle microhistological analysis (CMA) and PCR amplification combined with capillary electrophoresis (PCR-CE) in assessing diet composition of herbivores through faecal samples.

Days	Diet components	Weight (g)	Percentage (%)
1 to 7*	*Medicago sativa* (Ms)	*ad libitum*	
**8**	Diet 1:		
	*Calluna vulgaris* (Cv)	28.5	39.8
	*Carex caryophyllea* (Cc)	13.5	18.8
	*Festuca* spp.^1^	29.7	41.4
9* **to 12**	*Medicago sativa*	*ad libitum*	
**13**	Diet 2:		
	*Helianthemum nummularium* (Hn)	17.3	18.5
	*Juncus trifidus* (Jt)	6.7	7.2
	*Nardus stricta* (Ns)	47.0	50.3
	*Trifolium alpinum* (Ta)	22.5	24.1
14* **to 17**	*Medicago sativa*	*ad libitum*	
**18**	Diet 3:		
	*Arrhenatherum elatius* (Ae)	76.4	32.3
	*Hypochaeris radicata* (Hr)	68.7	29.2
	*Vaccinium uliginosum* (Vu)	89.4	38.5
19*	*Medicago sativa*	*ad libitum*	

Composition of the plant mixtures is indicated and the weight of each plant species in the mixture is given in grams (g) and in percentage (%). Plant species initials are listed between brackets.

* Days of faeces collection.

^1^ Includes a mixture of *F*. *eskia* (Fe), *F*. *gautieri* (Fg), *F*. *glauca* (Fgl), and *F*. *violacea* (Fv).

## Ethics statement

The approval to keep a captive Pyrenean chamois was supported by the Departament d’Agricultura, Ramaderia, Pesca, Alimentació I Medi Natural—Generalitat de Catalunya (DARPAMN—the Regional authority in charge of livestock and wildlife management). In this work we did not require the approval of the Ethical and Animal Welfare Committee of the Universitat Autònoma de Barcelona. In fact, the Directive 2010/63/EU of the European Parliament indicates: Specific protocols shall not apply to practices not likely to cause pain, suffering, distress or lasting harm equivalent to, or higher than, that caused by the introduction of a needle in accordance with good veterinary practice.

### Time-course degradation

Fresh faeces from the hunter-harvested Pyrenean chamois were randomly mixed in 4 pools and left outdoor at the Universitat Autònoma de Barcelona campus (Bellaterra, Spain) for three weeks starting in January 2016. This experiment was set up as a pilot assessment to evaluate the connection among the time-lapse between chamois deposition and faecal sampling—a potential degree of sample degradation—and the performance of both CMA and PCR-CE methods. In that period, temperatures ranged from 2.5°C to 21.7°C (mean = 11.1°C) with short periods of rain between weeks 2 and 3 (S1 Fig). Pellets from the four different mixtures were collected every week and kept at -20°C until processing. After three weeks of harvesting, they were subsequently tested as described below and the number of identified plant species was monitored using the two methods.

### Genomic DNA extraction and multiplex-PCR DNA amplification

Genomic DNA from plants and chamois faeces was extracted from 200 mg of raw material with the DNeasy Plant Mini Kit and the QIAamp DNA Stool Kit (Qiagen, Germany), respectively, following manufacturer’s instructions. Genomic DNA was eluted in 100 μl of milli-Q water and kept at -20°C. DNA concentration was measured with a NanoDrop 1000 Spectrophotometer (ThermoScientific, USA) and DNA quality was analysed on a 0.8% agarose gel electrophoresis in 1x TBE buffer.

Multiplex-PCR reactions for specific amplification of chloroplastic *trnL(UAA)* and nuclear *ITS2* sequences in plants and faeces were carried out in a total volume of 20 μL containing 1x MyTaq Reaction Buffer (Bioline Reagents Ltd, United Kingdom), 0.4 μM each oligonucleotide (Stab Vida, Portugal; see S1 Table for detailed oligonucleotide sequences), 1 U MyTaq HS DNA polymerase and 20 ng of genomic DNA template. Oligonucleotides trnL-G and trnL-D were used to amplify the *trnL(UAA)* region [47], while oligonucleotides S2F and S3R amplified the *ITS2* region [40]. Oligonucleotides trnL-D and S2F were fluorescence-labelled with 6-FAM (carboxyfluorescein) and HEX (hexachloro-6-carboxyfluorescein) fluorochromes, respectively, to allow simultaneous fluorescent detection by capillary electrophoresis.

Cycling conditions were optimized for multiplex DNA amplification and comprised an initial denaturation for 1 min at 95°C, followed by 35 cycles of denaturation for 15 s at 95°C, annealing for 15 s at 56°C and extension for 45 s at 72°C, and a final extension step for 3 min at 72°C. PCR reactions were run on a MJ Research PTC-100 Thermal Cycler (MJ Research Inc., Canada) and amplicons were visualised on a 1.5% agarose gel electrophoresis in 1x TBE buffer.

### DNA sizing

Amplicon lengths were determined by automated capillary electrophoresis with a Genetic Analyzer 3130xl system (Applied Biosystems, USA) using LizS600 as a size standard. Electropherograms were *in silico* analysed with provided Peak Scanner Software version 2.0 (Applied Biosystems, USA). Plant genomic sequences of the *trnL(UAA)* and *ITS2* regions were retrieved from the GenBank database using the nucleotide BLASTN tool (e-value < e-04) [50]. Sequence lengths were calculated by flanking corresponding primers to each plant sequence.

### Slide preparation for CMA analysis

Fresh faeces were lightly ground by hand in a mortar to separate out the epidermal fragments [51]. From each sample, 0.5 g of ground faeces were introduced into a test tube with 3 ml of concentrated HNO_3_ to allow non-epidermal tissue digestion. The test tubes were placed in a water bath at 80°C for 2 minutes and the samples were then diluted with 200 ml of distilled water. This suspension was successively sieved through 1 mm and 0.25 mm filters. The 0.25 to 1 mm fraction was dispersed in a 50% (v/v) aqueous solution of glycerine. Samples of the suspensions were spread on glass microscope slides at a density that prevented any significant overlapping of fragments. Cover slips (24 x 60 mm) were then fixed to the slides with DPX varnish (Herter Instruments, Spain). Three slides were prepared from each sub-sample. A reference epidermis collection of the different plant species included in the study was set up in parallel using two different methods. First, fresh plant material was coarsely ground by hand and then processed in the same way as the faeces samples in order to simulate the fragmentation of the epidermis during ingestion and digestion. Second, fresh abaxial and adaxial leaf surfaces were obtained with a scalpel and mounted on a glass microscope slide allowing a more accurate identification of the main morphological features of the different species.

### Fragment identification

Each slide was examined under a Motic BA210 optical microscope (MoticEurope SLU, Spain) at 400x magnification. Three traverses were scanned, each one 2 mm wide and 60 mm long with 3 mm between traverses. Up to 200 epidermis fragments were identified per sample using the reference collection of plant species, based on the shape of epidermal cells, trichomes and stomata surrounding cells. The data were then pooled and converted to percentages. Reference collection images were captured with a Moticam 2300 camera using provided Motic Images Plus 2.0 software.

### Statistical analysis

We used a linear model (LM) to explore the effects of the elapsed time between faecal deposition and sampling on the number of plant species identified (response variable) by CMA and PCR-CE. The LM included the sampling period (fixed categorical factor with four categories: Initial and 1^st^, 2^nd^, and 3^rd^ week) and the identification technique (CMA and PCR-CE) as fixed explanatory factors, and the number of identified plant species as a response variable. Due to our moderate sample size (n = 32, two techniques x four determinations per week), model parameter uncertainty was estimated by residual-based bootstrap methods using the Boot package 1.3–20 version [52]. LM assumptions were evaluated by exploring the residual pattern (homoscedasticity, linearity and normality) [53]. Weekly differences in the number of plants detected by both methodologies were tested by a Tukey’s HSD [54]. All statistical analyses were performed in R version 3.4.3 [55].

## Results

### DNA fragments length and CMA reference databases

We established a database of different plants, 13 of them representative of the eastern Pyrenees ecosystem and *Medicago sativa*, based on the size of multiplex-PCR amplified *trnL(UAA)* and *ITS2* genomic sequences with primers trnL-G/D and S2F/S3R, respectively. Both genes were successfully amplified in all the plants tested, confirming the universality of the oligonucleotides previously designed for plant DNA barcoding studies (S2 Fig) [40,47].

The size of the fluorescently labelled PCR products determined after capillary electrophoresis ranged from 303 to 593 bp for *trnL(UAA)* amplicons and from 463 to 500 bp for *ITS2* amplicons, which strongly match the lengths calculated from retrieved GenBank sequences (S3 Fig and Table 2). Despite the fact that *trnL(UAA)* exhibits higher resolution than *ITS2*, since the corresponding PCR products show a wider range of sizes, the data obtained from the two genomic sequence amplifications is complementary. Thus, *Calluna vulgaris* (L.) Hull, *Carex caryophyllea* Latourr. and *Vaccinium uliginosum* L. showed similar *ITS2* fragment sizes (488, 490 and 492 bp, respectively), but they could be clearly discriminated through their *trnL(UAA)* fragments (492, 593 and 464 bp, respectively). The species of the genus *Festuca* were almost indistinguishable by their *ITS2* fragments (472–475 bp) but exhibited distinct *trnL(UAA)* fragment sizes (504–517 bp), with the exception of *F*. *eskia* Ramond ex DC. and *F*. *gautieri* (Hack.) K.Richt., which were identical. Furthermore, *Nardus stricta* L. and *Trifolium alpinum* L. could be clearly differentiated by their *ITS2* fragment (471 and 480 bp, respectively), better than by the corresponding *trnL(UAA)* fragment (518 and 516 bp). Three species, *Arrhenatherum elatius* (L.) P. Beauv. ex J. Presl & C. Presl, *M*. *sativa* and *Juncus trifidus* L., exhibited a shorter *trnL(UAA)* fragment, which sharply differed from the rest of the plant species.

**Table 2:** DNA fragment lengths reference database for the plant species included in the controlled diet test.

Plant species	*trn*L(UAA)			ITS2		
	amplicon size (bp)	fragment sequence length (bp)	GenBank accession	amplicon size (bp)	fragment sequence length (bp)	GenBank accession
*Arrhenatherum elatius*	303	310	EU434100	471	NA	-
*Calluna vulgaris*	492	495	KP737377	488	497	KP737514
*Carex caryophyllea*	593	593	EU288467	490	499	HG915830
*Festuca eskia*	517	519	AF478508	473	484	KF917344
*Festuca gautieri*	517	519	KP699267	472	482	AF303414
*Festuca glauca*	504	506	JX871940	475	485	AY327792
*Festuca violacea*	508	510	EF593012	472	484	EF584979
*Helianthemum nummularium*	491	NA	-	463	479	GU327669
*Hypochaeris radicata*	530	532	AY504774	483	485	AF528461
*Juncus trifidus*	343	345	AY437971	500	495	AY973508
*Medicago sativa*	326	327	GQ488614	484	486	AF053142
*Nardus stricta*	518	520	EU434097	471	479	KJ477049
*Trifolium alpinum*	516	522	DQ311725	480	484	DQ311995
*Vaccinum uliginosum*	464	466	DQ860640	492	498	DQ217769

Amplicon sizes were determined by capillary electrophoresis in this study. Fragment sequence lengths were calculated from retrieved GenBank sequences. (bp), base pairs. NA, not available.

**Fig 1:**
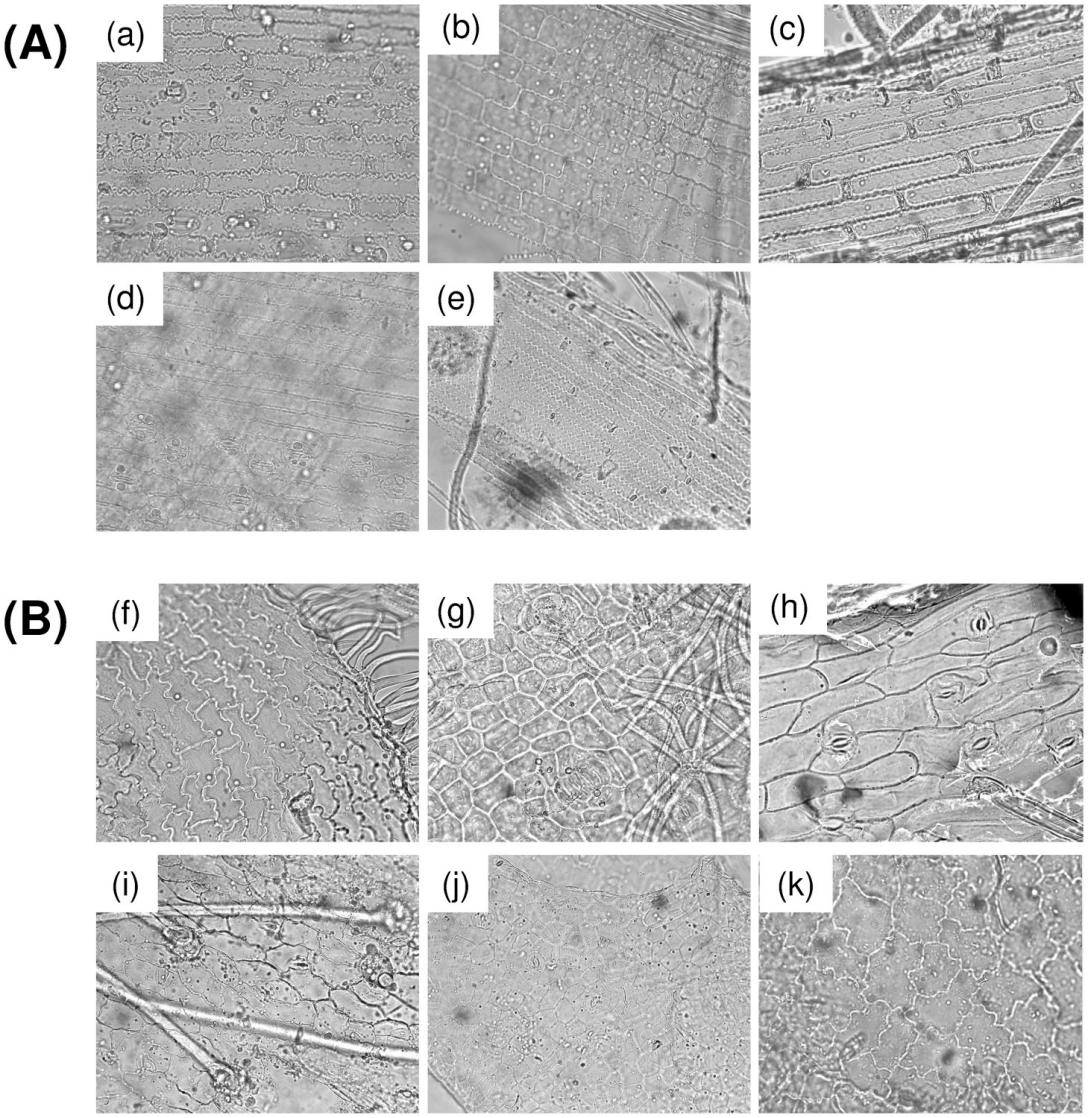
Microscopical images showing the main epidermal anatomical features of the plant species used in this study. (A) Graminoid species: (a) *Arrhenatherum elatius*, (b) *Carex caryophyllea*, (c) *Festuca* spp., (d) *Juncus trifidus*, (e) *Nardus stricta*; (B) Dicotyledonous species: (f) *Calluna vulgaris*, (g) *Helianthemum nummularium*, (h) *Hypochaeris radicata*, (i) *Medicago sativa*, (j) *Trifolium alpinum*, (k) *Vaccinium uliginosum*. Images were obtained under 400 x magnification.

Concerning CMA, all plant epidermis fragments were easily distinguishable at the genus level based on their anatomical structures [56]. In summary, graminoids presented rectangular epidermic cells but differences between the species in terms of cell size, cell wall shape and the presence and shape of trichomes and stomata (Fig 1A). *C*. *cariophyllea* fragments presented square to rectangular epidermic cells with wavy cell walls, prickle-shape trichomes and diacytic stomata. *J*. *trifidus* also presented rectangular elongated epidermal cells but the wall was smoother. *A*. *elatius* displayed rectangular epidermic cells with wavy walls intercalated with short cells. *N*. *stricta* also had rectangular epidermis cells with wavy walls but were longer than *A*. *elatius* and some prickle-shaped trichomes could also be observed. *Festuca* spp. showed long epidermic cells with wavy walls separated by C-shaped short cells, paracytic stomata and spindle-shaped trichomes. Depending on the *Festuca* species, cell walls displayed variable waviness, yet fragments could not be characterised at the species level due to the extraordinary morphological similarity exhibited by this genus.

Dicots were also clearly distinguishable by their specific micro-anatomical features, especially trichomes and stomata (Fig 1B). For Ericaceae, *C*. *vulgaris* presented lobed epidermal cells and anisocytic stomata protected by medium-sized, wavy trichomes while *V*. *uliginosum* displayed angular to lobed epidermal cells, paracytic stomata and no trichomes. Additionally, veins were easily observable in the *V*. *uliginosum* fragments. *Helianthemum nummularium* (Cav. Losa & Rivas Goday) showed angular polygonal epidermal cells, cyclocytic stomata and star-shaped trichomes. *Hypochaeris radicata* L. presented irregular rectangular cells with angles often different than 90 degrees, anamocytic stomata and large multicellular hairs. Finally, Fabaceae *T*. *alpinum* fragments showed angular polygonal epidermal cells, anomocytic stomata and medium-sized trichomes with pointed tips, and *M*. *sativa* displayed polygonal epidermal cells with undulated cell walls and long single-cell trichomes with a reflective dual-cell base.

### Analysis of the botanical composition of chamois faeces subjected to a controlled diet

**Fig 2:**
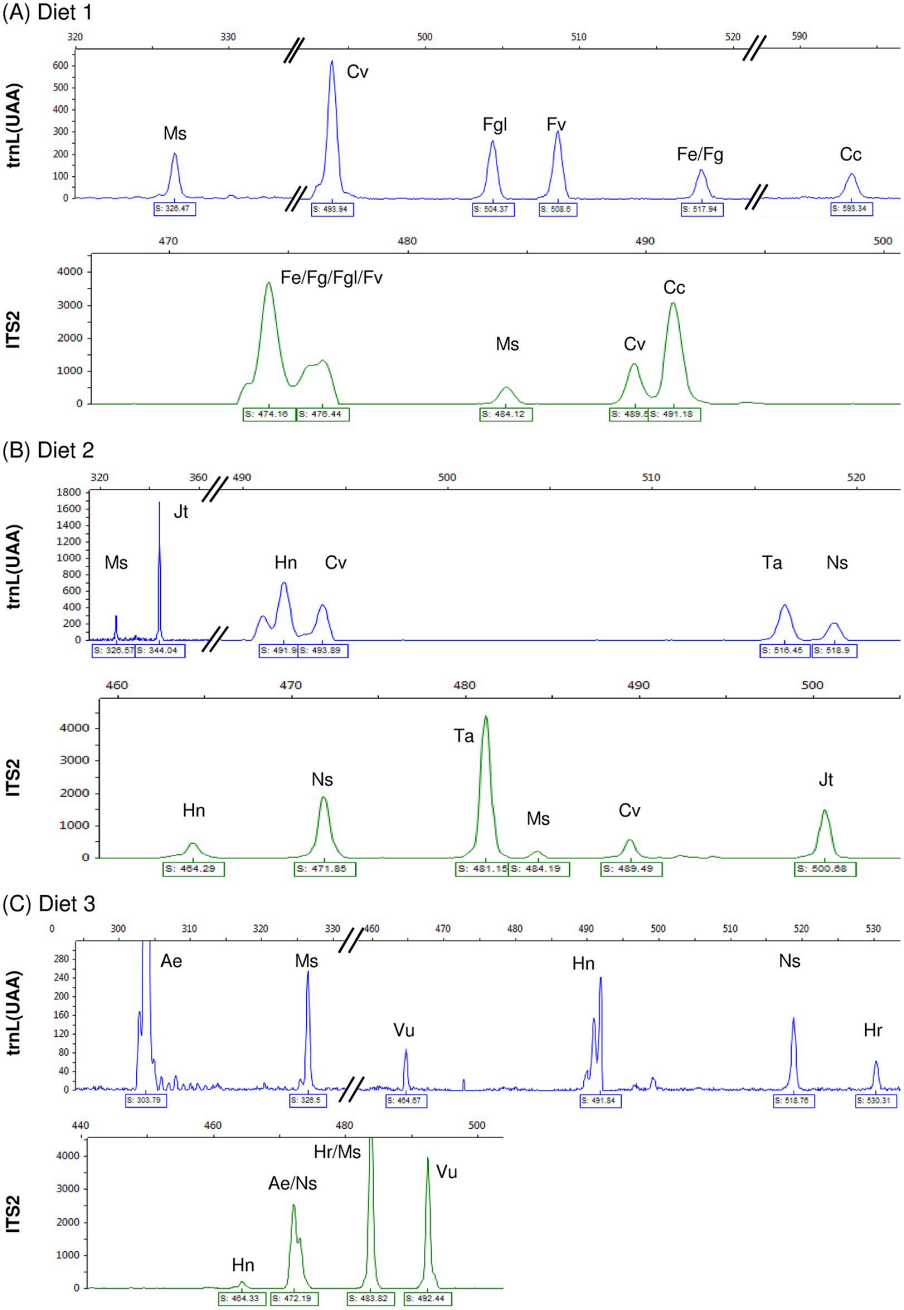
Electropherograms peaks of the three controlled diets offered to the stabled Pyrenean chamois, obtained by capillary electrophoresis of the fluorescence labelled PCR products corresponding to *trnL(UAA)* (blue) and *ITS2* (green) multiplex amplification. For a better interpretation, the two fluorescence channels are shown separately. Plant species initials are as in Table 1.

The 13 plant species that were distributed in three different mixture diets and offered to a Pyrenean chamois were completely ingested within the timeframe they were offered. Multiplex PCR amplification of *trnL(UAA)* and *ITS 2* sequences from genomic DNA extracted from chamois faeces yielded products of expected size in all the samples tested (S4 Fig). Analysis of fluorescently labelled PCR products by capillary electrophoresis showed precise and independent peaks of different lengths that could be roughly ascribed to a particular plant species (Fig 2). We were able to identify all the plants included in the controlled diets. For a better interpretation of the electropherograms, the two fluorescence channels were independently preselected. Two common peaks among the three controlled diets of 326 and 484 bp for *trnL(UAA)* and *ITS2* amplicons, respectively, were observed and would correlate with *M*. *sativa* background intake.

In diet 1 electropherograms, 6 peaks for *trnL(UAA)* and 5 peaks for *ITS2* amplicons were distinguished, which may correspond to the 7 plant species included in the diet (Fig 2A). In contrast, two species of the genus *Festuca*, *F*. *eskia* and *F*. *gautieri* remained undetermined. A wider peak of 474–476 bp in the *ITS2* amplification was assigned to the *Festuca* genus. Diet 2 electropherograms exhibited 7 peaks for *trnL(UAA)* and 6 peaks for *ITS2* amplicons that could be assigned to the 4 diet species (Fig 2B). One *trnL(UAA)* peak, adjacent to *H*. *nummularium*, did not match any plant species but may match a particular amplification profile for this species (S3 Fig). Furthermore, one of the peaks for each gene correlated with *C*. *vulgaris* size amplicons suggesting that residual genomic DNA of this species belonging to diet 1 was present in the analysed faeces. Moreover, diet 3 *trnL(UAA)* electropherograms displayed a total number of 6 peaks: 3 of them corresponded to the diet species, the other 2 to residual *H*. *nummularium and N*. *stricta* from diet 2, and the common *M*. *sativa* peak. For the *ITS2* channel, results were less resolved as two of the peaks (472 and 483 bp) matched more than one species (Fig 2C).

Regarding CMA analysis, all the species included in the study were identified and quantified in the faeces, with the exception of the species of the genus *Festuca*, which could not be discriminated due to their almost identical epidermal structures. Results are given as percentages of epidermal fragments quantified in faecal samples (Table 3). Quantification of *M*. *sativa* was discarded for better determination of experimental plants. Our results show that epidermal fragments from the different species were preserved in the successive faecal samples throughout the experiment to the extent that species included in diets 1 and 2 were also detected in diet 3.

**Table 3:** Percentages of epidermal plant fragments quantified in faecal samples (EPF) collected from a stabled specimen of Pyrenean chamois and determined by cuticle microhistological analysis.

Diet 1	EPF (%)	±	SE	Diet 3	EPF (%)	±	SE
*Calluna vulgaris*	74.8		4.4	*Arrhenatherum elatius*	18.7		4.2
*Festuca* spp.	16.1		3.3	*Nardus stricta*	17.6		5.6
*Carex caryophyllea*	9.1		0.7	*Festuca* spp.	14.9		3.5
				*Hipochaeris radicata*	10.9		3.1
Diet 2	EPF (%)	±	SE	*Vaccinum uliginosum*	9.4		1.9
*Helianthemum num*.	28.3		2.9	*Helianthemum num*.	8.8		1.5
*Trifolium alpinum*	27.8		6.5	*Calluna vulgaris*	7.5		3.3
*Calluna vulgaris*	16.6		3.6	*Juncus trifidus*	4.9		0.7
*Nardus stricta*	9.7		2.3	*Carex caryophyllea*	4.5		1.0
*Festuca* spp.	9.4		1.6	*Trifolium alpinum*	2.9		1.7
*Juncus trifidus*	7.9		1.2				
*Carex caryophyllea*	0.3		0.3				

(SE), standard error.

Furthermore, the CMA approach enables the possibility of linking percentages of epidermal fragments quantified in faeces (% EPF) with those of plant ingested weights (% I). Thus, an EPF/I ratio can be defined for each species in the corresponding diet, providing information about a putative over-detection of a particular species in faeces when EPF/I > 1 (Table 4). For a simplification of the model, only original diet species have been included in ratio calculations.

**Table 4:** Ratios of epidermal plant fragments (EPF) quantified by cuticle microhistological analysis (CMA) in faecal samples from a stabled specimen of Pyrenean chamois *versus* ingested plant weights (I) for each plant species in the corresponding diet.

	Plant species	Herbaceous/shrub	% ingest (I)	% EPF (EPF)	EPF/I	Lignin (% of dry matter)	Ref.
**Diet 1**	*Calluna vulgaris*	Shrub	39.8	74.8	1.9	17.8	[57]
	*Carex caryophyllea*	Herbaceous	18.8	9.1	0.5	3.3	[58]
	*Festuca* spp.	Herbaceous	41.4	16.1	0.4	5.4	[14]
**Diet 2**	*Helianthemum num*.	Shrub	18.5	28.3	1.5	7.4	[59]
	*Juncus trifidus*	Herbaceous	7.2	7.9	1.1	2.8	[60]
	*Nardus stricta*	Herbaceous	50.3	9.7	0.2	5.4	[58]
	*Trifolium alpinum*	Herbaceous	24.1	27.8	1.1	6.9	[58]
**Diet 3**	*Vaccinum uliginosum*	Shrub	38.5	9.4	0.2	7.4	[61]
	*Arrhenatherum elatius*	Herbaceous	32.3	18.7	0.6	ND	-
	*Hypochaeris radicata*	Herbaceous	29.2	10.9	0.4	3.1	[62]

Overdetection is reached when EPF/I >1.

Lignin content values were taken from the indicated references. ND, data not reported in the bibliography.

For example, in diet 1, ingested quantities of *C*. *vulgaris* and *Festuca* spp were equivalent and doubled that of *C*. *caryophyllea* ingests (39.7%, 41.4% and 18.8%, respectively) but the first was the predominant species detected in faeces (74.8%, 16.1% and 9.1% resp.). EPF/I ratios suggest an over-detection of *C*. *vulgaris* (EPF/I = 1.9) and a significant equivalent infra-detection of *Festuca* spp (EPF/I = 0.4) and *C*. *caryophyllea* (EPF/I = 0.5). A similar behaviour is observed in diet 2. Despite the fact that *H*. *nummularium* was not the most ingested species, it was over-detected in the corresponding faeces (EPF/I = 1.5), while *N*. *stricta*, which was the main species in the diet, was poorly detected, showing a EPF/I ratio of 0.2, whereas for *J*. *trifidus* and *T*. *alpinum* the EPF/I was 1.1. The three plant species included in diet 3 were infra-detected in faeces showing EPF/I ratios of 0.2–0.6.

### Time-course degradation analysis

Descriptive statistics for the mean number of plant species found in our pool of faecal samples exposed to outdoor climatic conditions is shown in Table 5. According to our LM, 94.4% of the observed variability in the number of plant species detected was explained by the interaction between the time elapsed since samples were exposed to the environment and the detection technique (F_7, 14_ = 40.5, p < 0.001). The mean number of plant species detected by PCR-CE was notably higher than the mean number detected by CMA at the initial starting point of outdoor exposure. However, while the number of species identified by CMA was fairly stable during the trial, the species detected by the molecular method dropped by more than half since the first week. In the faecal material exposed to the environment for three weeks, no species were detected by this technique. According to the Tuckey’s HSD test, the mean number of detected species by both techniques differed significantly in all sampling periods except for the initial sampling (*q* = -2.25 [-4.73;0.23], *p* = 0.09, Table 5).

**Table 5:** Mean ± standard deviation, minimum and maximum number of plant species detected in a pool of Pyrenean chamois faeces exposed to outdoor conditions using cuticle microhistological analysis (CMA) and PCR amplification combined with capillary electrophoresis (PCR-CE).

Sampling period	CMA	PCR-CE
**Initial**	8.75 ± 0.5 (8–9)	11 ± 1.83 (9–13)
**1^st^ Week**	8.75 ± 1.5 (7–10)	4.25 ± 0.71* (3–5)
**2^nd^ Week**	8.75 ± 0.96 (8–10)	1 ± 0.82* (0–2)
**3^rd^ Week**	8 ± 0.82 (8–10)	0 ± 0* (--)

* denotes significant differences between methods, at α = 0.05, Tukey’s HSD test.

## Discussion

In this article, we describe and compare two technical approaches for the study of the diet of herbivores (folivores *sensu stricto*) using faecal samples as a source for analysis: a modified PCR-based DNA amplification of marker genes (PCR-CE approach) and a microhistology-based identification of the remaining plant epidermis (CMA approach). In both methodologies, a previous description of the plant species that constitute the available resources for herbivores is an essential prerequisite. The identification of the putative ingested plant species is supported by the collation of the information obtained from the remaining plant epidermis and/or genomic DNA in the faeces with those available in plant reference databases.

Concerning the molecular approach, we have developed an optimized method for multiplex-PCR DNA amplification of two genomic sequences including chloroplast *trnL(UAA)* and nuclear *ITS2* plant genes using total genomic DNA extracted from herbivore faeces as a template. The use of fluorescently labelled oligonucleotides enables the subsequent determination of the amplicon sizes by capillary electrophoresis, which in turn is the basis for plant species determination in diet composition. It has been previously described that DNA barcoding of *trnL(UAA)* using conserved oligonucleotides G and H broadly amplifies a fragment of 10–143 bp covering the P6 loop of the plant *trnL(UAA)* intron, and plant species adscription relies on the application of massive sequencing technologies [30,47]. We show in this work that the amplification of larger *trnL(UAA)* fragments (310–593 bp), including the conserved oligonucleotides G and D and comprising the intron and the exon 2 gene regions, greatly improves the discriminating capacity of the technique due to the hypervariable length of the region and enables a more accurate identification of plant species. Moreover, the Internal Transcribed Spacer 2 (*ITS2*) region, situated between the 5.8S and 26S genes of the nuclear ribosomal cistron, has also been considered an optimal candidate gene marker, due to its appreciable interspecific divergences even in closely related species [39,40]. Since the unique plant structures that can be distinguished in faeces samples are the epidermal cells, which lack chloroplast organelles, the concomitant amplification of a nuclear genomic sequence, such as *ITS2*, serves as a control for DNA template quality. In addition, *ITS2* analysis supplements the data obtained from *trnL(UAA)* concerning plant species identification belonging to a particular genus (i.e., *Festuca* genus), suggesting that the information obtained from both genes may be complementary. Furthermore, genomic sequences of *trnL(UAA)* and *ITS2* genes from many plant species are freely accessible through public databases, such as GenBank [43], enabling the *in silico* prediction of gene lengths. It is noteworthy that retrieved GenBank sequences corresponding to both genes are a few base pairs longer compared to the fragment amplicon sizes experimentally determined, probably due to the intrinsic sensitivity of the capillary electrophoresis procedure, as previously described [32]. However, these slight variations do not affect the identification of the plant species since fragment sizes determined by capillary electrophoresis are equivalent in fresh plant tissues and in faeces samples analysis. Although we tested the efficacy of PCR-CE combining two complementary genes on faecal samples from free-ranging chamois, the reliability and resolution of the method should be tested on even more species-diverse diets [14].

CMA provides valuable information about herbivore diet choices through microscopic recognition of digested plant epidermises [16,51]. This method has been widely used for decades due to its versatility and low economic cost, although a high degree of observer expertise is required for accurate microscope interpretation and the level of discrimination between some related plants is relatively low [63]. Microhistological analysis of faeces is more precise than macroscopic identifications of rumen contents [46,64], and may serve to assess diet composition not only in game ungulates but also in species with some degree of protection (e.g., Tatra or Abruzzi chamois among others), thus offering the opportunity to avoid invasive hunting praxis. One of the strong features of CMA is that it offers the possibility to perform semi-quantitative determinations of herbivore diet composition (folivores *sensu stricto*), through the direct extrapolation of the plant epidermal fragment quantities present in the faecal samples [20]. However, differing degrees of digestibility of plant species that may determine the remaining plant material in the faeces must be considered [18,57,65,66]. To explain this apparent inconsistency, we propose an index of preservation for each plant species, called EPF/I ratio, that would link the relative quantity of one species detected in a faecal sample (EPF) to the corresponding ingested quantity (I). The index would reflect a putative over-detection of a plant species in the faeces when EPF/I > 1, due to a slow or a reduced digestion process of the ingested plant tissues. Considering the plant species included in the study, the shrubby or herbaceous constitution of the plant is most likely the main factor determining the digestibility of a particular plant species. The plant cell wall is composed of polysaccharides and secondary metabolites that assemble to generate complex structures. Shrubs are persistent woody plants with reinforced and thicker cell walls compared to herbaceous plants, partially due to their high lignin content, and therefore are prone to a relatively low digestibility [17,58,60]. Intriguingly, the lignin content values reported in the bibliography for the species included in this study, describe an overall correlation with their structural constitution, where the highest lignin contents are consistent with the shrubby species—*C*. *vulgaris* and *H*. *nummularium*—and, concomitantly, with their average over-detection in faeces (Table 4). This observation suggests that lignin may exert a protective effect over plant structures throughout the digestion process, which would lead to their preservation, thus triggering the over-detection of shrubby compared to herbaceous species in the faeces. However, lignin content and plant constitution may not be the only explanations for the discrepancies between plant ingests and plant fragments detected in faeces (EPF/I ≠ 1), since in our experimental conditions two herbaceous species, *J*. *trifidus* and *T*. *alpinum*, showed equivalent EPF/I ratios, but different lignin contents. It is also notable that the concomitant detection of many plant species hinders the observation under the microscope and debases the relative species quantification, thus hampering the conclusive establishment of EPF/I correlations. This fact occurred in the corresponding faecal samples from diet 3 and especially in the case of the shrubby *V*. *uliginosum*. We propose that further determinations of individual EPF/I ratios for preferred plant species would provide valuable and more reliable information in CMA-based herbivore diet studies.

The greater resistance to digestion of some shrubby plant species observed by CMA was also perceived at the molecular level since *C*. *vulgaris* (diet 1) and *H*. *nummularium* (diet 2) were PCR-amplified in faecal samples belonging to diets 2 and 3, respectively, while none of the herbaceous plants showed this behaviour. Furthermore, PCR amplification is roughly independent of the structural constitution of a plant species but strongly hinges on sample freshness. Thus, while the number of species detected by CMA remained stable in samples kept outdoors for three weeks, the number of species amplified by PCR markedly decayed within the first week. The length of the PCR amplicons, which were 450 bp on average, provides high resolution to discriminate between plant species, but the progressive degradation of the genomic DNA due to deficient preservation conditions is a drawback for amplification success [25,32,48,67]. Although PCR amplification of shorter genomic fragments (70–100 bp in length) from ancient samples containing partially degraded genomic DNA has been previously described in DNA barcoding studies, plant species adscription was performed through massive DNA sequencing instead of amplicon length determination [26–28]. Massive sequencing technologies are powerful tools that provide accurate information but require expensive technology and specific bioinformatic expertise that can serve as a barrier to standard implementation. We propose that determination of amplicon sizes by capillary electrophoresis is a precise, less time-consuming, standardisable and affordable method and that it can replace complex massive sequencing technologies in routine studies of diet composition from fresh faecal samples, as has been previously pointed out [32,42]. As a general guideline, we recommend to start performing a botanical assessment of the study area to not only create a reference collection for CMA but also retrieve the corresponding species genomic sequences from the GenBank database. Then, if researchers are only interested in a qualitative assessment, PCR-CE should be favoured due to its lower time consumption and its higher accuracy to detect plants at the species level (e.g. *Festuca* spp.). On the other hand, CMA must be used when a quick, easy and unexpensive semi-quantitative assessment on field collected faecal samplesis required [68].

## Conclusions

Taken together, our results suggest that PCR-CE combining the simultaneous amplification of two genes—long *trnL(UAA)* and *ITS2*—is a reliable technique for the determination of diet composition in herbivores. However, while this DNA-based method is qualitative in our experimental conditions, CMA can provide semi-quantitative data, since individual percentages of ingested plant species can be calculated, although a differential index of plant preservation should be taken into account. Additionally, the experimental conditions of outdoor exposure acted concomitantly with proper digestion processes to trigger genomic DNA degradation. Consequently and as expected, faeces freshness and preservation conditions are critical and determinant factors that compromise downstream DNA amplification efficiency but are not limiting in the case of CMA. We conclude that CMA and PCR-CE have both strong and weak features that make the two approaches complementary. Therefore, their simultaneous use can provide more complete, accurate and thorough information in diet composition studies. However, neither CMA nor PCR-CE seems to solve comprehensively the quatification of herbivore diets and thus further research needs to be done.

## Supporting information

S1 Dataset Raw data. (XLSX)

S1 Fig **(A) Picture of the four pools of faecal samples from the time-course degradation experiment**. **(B) Environmental conditions while faecal samples where left outdoor** (red line: mean daily temperature; red dotted line: minimum daily temperature; red dashed line: maximum daily temperature; blue bars: daily cumulative rainfall).
(TIF)

S2 Fig Migration patterns on a 1.5% agarose electrophoresis of *trnL(UAA)* and *ITS2* PCR products corresponding to the reference plant database amplifications. Ae: *Arrhenatherum elatius*, Cv: *Calluna vulgaris*, Cc: *Carex caryophyllea*, Fe: *Festuca eskia*, Fg: *Festuca gautieri*, Fgl: *Festuca glauca*, Fv: *Festuca violacea*, Hn: *Helianthemum nummularium*, Hr: *Hypochaeris radicata*, Jt: *Juncus trifidus*, Ms: *Medicago sativa*, Ns: *Nardus stricta*, Ta: *Trifolium alpinum*, Vu: *Vaccinum uliginosum*. (-): non template negative PCR control. 1Kb ladder is a reference ladder marker.
(TIF)

S3 Fig Electropherogram peaks of the 14 plant species included in this study, obtained after capillary electrophoresis of the fluorescense labelled PCR products corresponding to *trnL(UAA)* (blue) and *ITS2* (green) multiplex amplification. Peak sizes are summarized in Table 2.
(TIF)

S4 Fig Migration patterns on a 1.5% agarose electrophoresis of *trnL(UAA)* and *ITS2* PCR products amplified from previous controlled diet faeces genomic DNA (*Medicago sativa* intake, Ms) and from faeces genomic DNA corresponding to diets 1 to 3. Composition of diets is detailed in Table 1. (-): non template negative PCR control. 1Kb ladder is a reference ladder marker.
(TIF)

S1 Table Universal oligonucleotide sequences used for specific PCR amplification of chloroplastic *trnL(UAA)* and nuclear *ITS2* regions. Fluorescence-labelling of oligonucleotides TRNL_D and S2F is indicated. Expected amplicon size is given in base pairs (bp).
(DOCX)
