# Supplementary figures and images for: Comparing the accuracy of PCR-capillary electrophoresis and cuticle microhistological analysis for assessing diet composition in ungulates: A case study with Pyrenean chamois
Source: PLoS One. 2019 May 22;14(5):e0216345. doi: 10.1371/journal.pone.0216345 (PMC6530829; doi:10.1371/journal.pone.0216345)

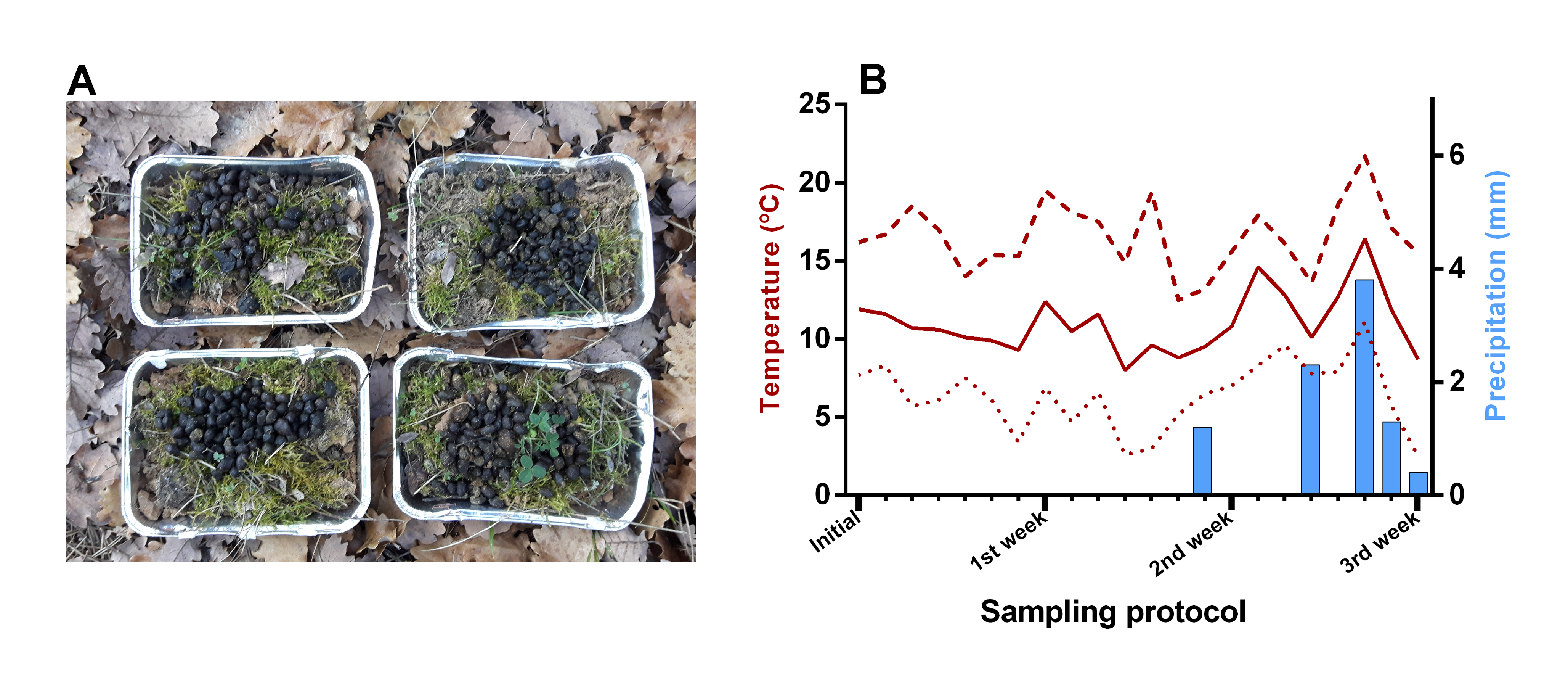

Supplement: S1 Fig — (A) Picture of the four pools of faecal samples from the time-course degradation experiment. (B) Environmental conditions while faecal samples where left outdoor (red line: mean daily temperature; red dotted line: minimum daily temperature; red dashed line: maximum daily temperature; blue bars: daily cumulative rainfall). (TIF) [file pone.0216345.s002.tif]

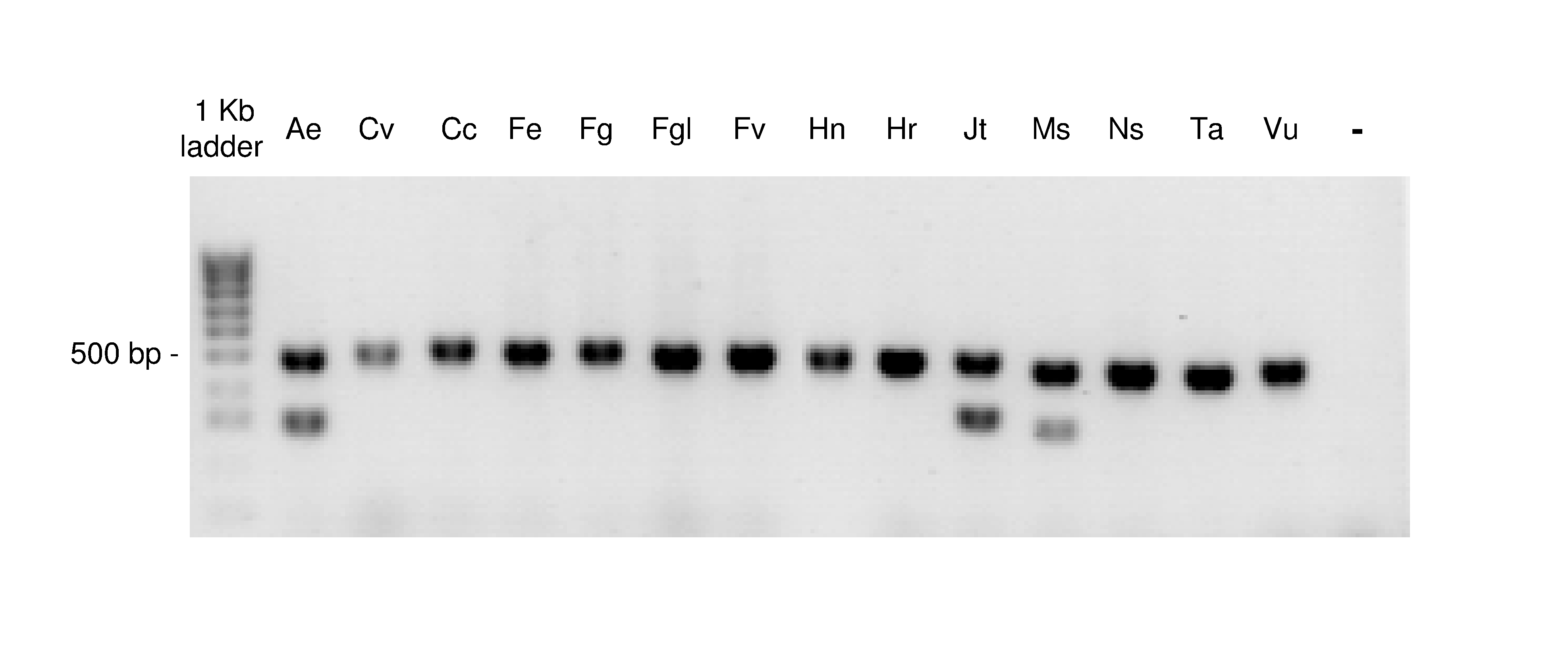

Supplement: S2 Fig — Ae: Arrhenatherum elatius, Cv: Calluna vulgaris, Cc: Carex caryophyllea, Fe: Festuca eskia, Fg: Festuca gautieri, Fgl: Festuca glauca, Fv: Festuca violacea, Hn: Helianthemum nummularium, Hr: Hypochaeris radicata, Jt: Juncus trifidus, Ms: Medicago sativa, Ns: Nardus stricta, Ta: Trifolium alpinum, Vu: Vaccinum uliginosum. (-): non template negative PCR control. 1Kb ladder is a reference ladder marker. (TIF) [file pone.0216345.s003.tif]

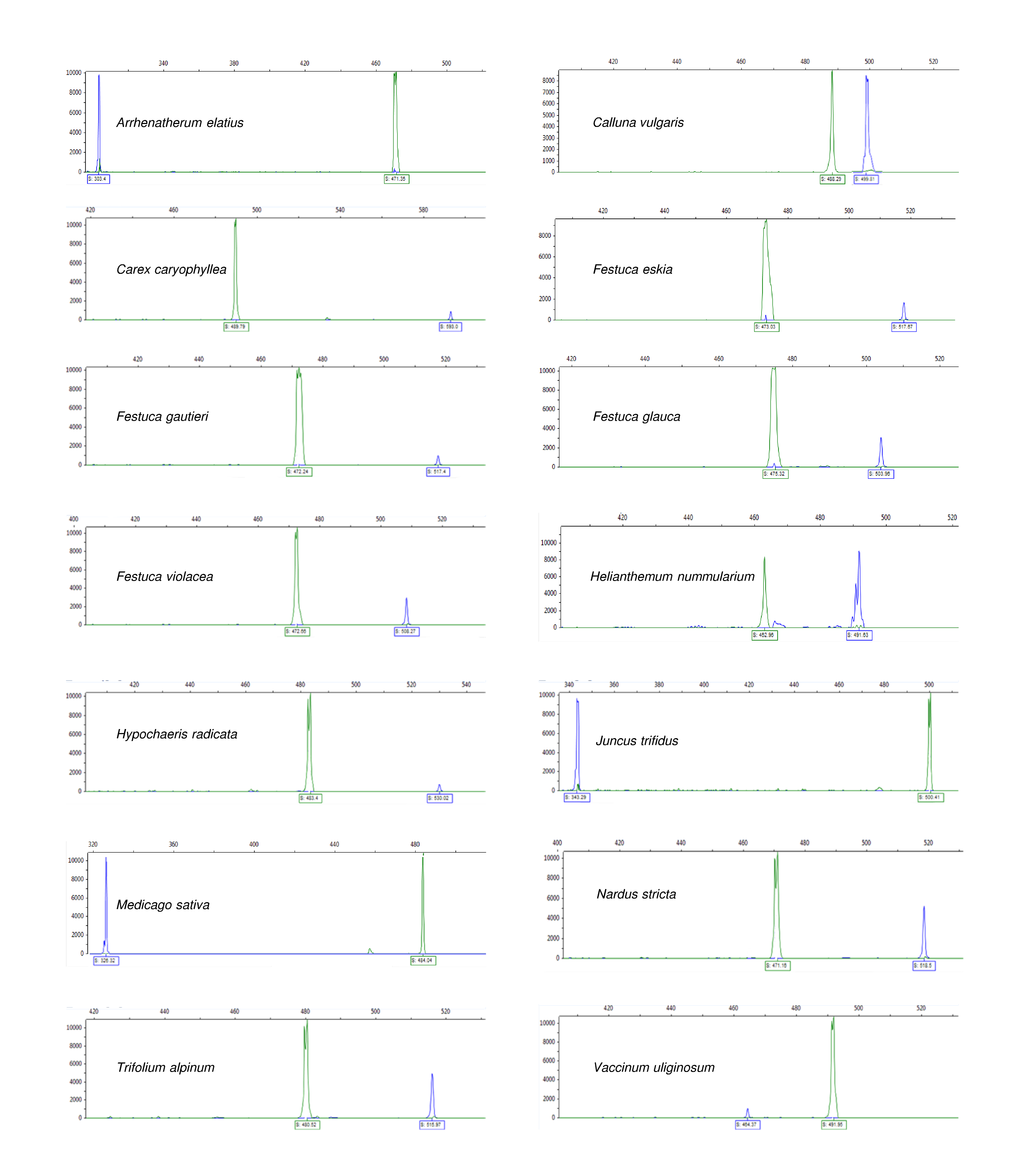

Supplement: S3 Fig — Peak sizes are summarized in Table 2. (TIF) [file pone.0216345.s004.tif]

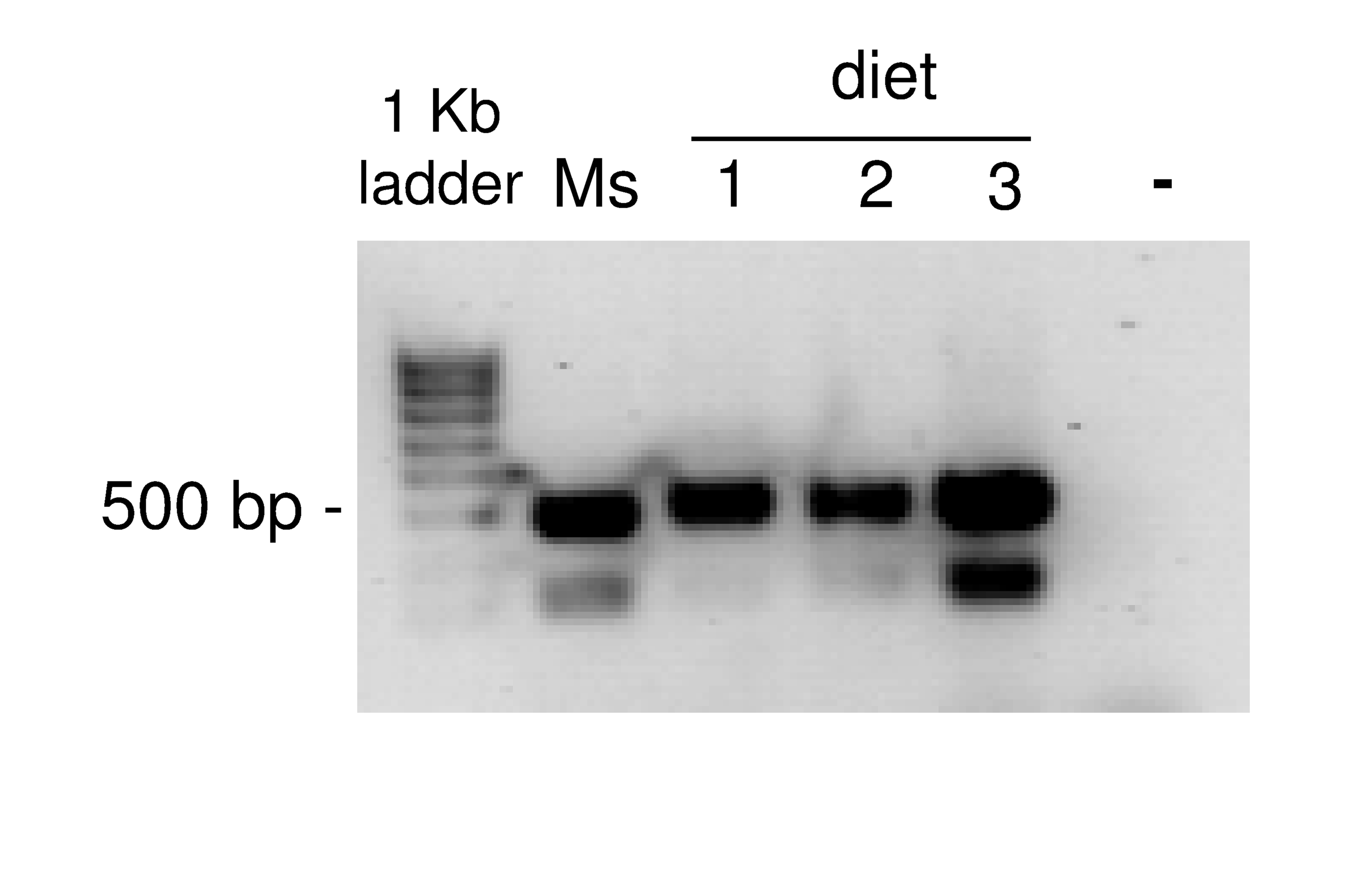

Supplement: S4 Fig — Composition of diets is detailed in Table 1. (-): non template negative PCR control. 1Kb ladder is a reference ladder marker. (TIF) [file pone.0216345.s005.tif]
