# Supplementary material for: Comparing the accuracy of PCR-capillary electrophoresis and cuticle microhistological analysis for assessing diet composition in ungulates: A case study with Pyrenean chamois
Source: PLoS One. 2019 May 22;14(5):e0216345. doi: 10.1371/journal.pone.0216345 (PMC6530829; doi:10.1371/journal.pone.0216345)
Supplement: S1 Table — Fluorescence-labelling of oligonucleotides TRNL_D and S2F is indicated. Expected amplicon size is given in base pairs (bp). (DOCX) [file pone.0216345.s006.docx]

|  | **Sequence** | **Position** | **Target genomic sequence** | **Fluorochrome dye** | **Amplicon size (bp)** | **Reference** |
| --- | --- | --- | --- | --- | --- | --- |
| **TRNL_G** | ^5’^gggcaatcctgagccaaatc^3’^ | Forward | *trnL(UAA)* | - | 310-593 | Taberlet (2007) and GenBank |
| **TRNL_D** | ^5’^ggggatagagggacttgaac^3’^ | Reverse |  | 6-FAM |  |  |
|  |  |  |  |  |  |  |
| **S2F** | ^5’^atgcgatacttggtgtgaat^3’^ | Forward | *ITS2* | HEX | 195-510 | Chen  (2010) |
| **S3R** | ^5’^gacgcttctccagactacaat^3’^ | Reverse |  | - |  |  |
